# UWB Radio-Based Motion Detection System for Assisted Living

**DOI:** 10.3390/s21113631

**Published:** 2021-05-23

**Authors:** Klemen Bregar, Andrej Hrovat, Mihael Mohorčič

**Affiliations:** Jožef Stefan Institute, Jamova 39, 1000 Ljubljana, Slovenia; klemen.bregar@ijs.si (K.B.); andrej.hrovat@ijs.si (A.H.)

**Keywords:** motion detection, assisted living, ultra-wideband, channel impulse response, power delay profile

## Abstract

Because of the ageing population, the demand for assisted living solutions that can help prolonging independent living of elderly at their homes with reduced interaction with caregivers is rapidly increasing. One of the most important indicators of the users’ well-being is their motion and mobility inside their homes, used either on its own or as contextual information for other more complex activities such as cooking, housekeeping or maintaining personal hygiene. In monitoring users’ mobility, radio frequency (RF) communication technologies have an advantage over optical motion detectors because of their penetrability through the obstacles, thus covering greater areas with fewer devices. However, as we show in this paper, RF links exhibit large variations depending on channel conditions in operating environment as well as the level and intensity of motion, limiting the performance of the fixed motion detection threshold determined on offline or batch measurement data. Thus, we propose a new algorithm with an online adaptive motion detection threshold that makes use of channel impulse response (CIR) information of the IEEE 802.15.4 ultra-wideband (UWB) radio, which comprises an easy-to-install robust motion detection system. The online adaptive motion detection (OAMD) algorithm uses a sliding window on the last 100 derivatives of power delay profile (PDP) differences and their statistics to set the threshold for motion detection. It takes into account the empirically confirmed observation that motion manifests itself in long-tail samples or outliers of PDP differences’ probability density function. The algorithm determines the online threshold by calculating the statistics on the derivatives of the 100 most recent PDP differences in a sliding window and scales them up in the suitable range for PDP differences with multiplication factors defined by a data-driven process using measurements from representative operating environments. The OAMD algorithm demonstrates great adaptability to various environmental conditions and exceptional performance compared to the offline batch algorithm. A motion detection solution incorporating the proposed highly reliable algorithm can complement and enhance various assisted living technologies to assess user’s well-being over long periods of time, detect critical events and issue warnings or alarms to caregivers.

## 1. Introduction

An increasing number of elderly people in developed countries want to or have to spend their late years living as independently as possible. In order to support this desire and also to assist professional and voluntary caregivers, modern assisted living (AL) technology solutions can be employed to support and ease everyday lives of the elderly, to prolong the time they are living independently, and, on the other hand, to also spare caregivers from unnecessary and routine checks on elderly and give them an opportunity to use their time when and where actually needed and for higher-quality interaction.

In the past decade, an increasing number of technological solutions started to emerge for supporting everyday lives of elderly and disabled people. Some of the areas where the AL systems can assist the living include personalized coaching, activity tracking, health parameters tracking, detecting emergency situations, etc. Among the most important parameters indicating the user’s well-being is their motion and mobility, which can be tracked by several technologies and also used as additional contextual information for other services and solutions. Motion and activity detection are crucial in healthcare application since the daily activities of elderly and their patterns represent vital information for early identification of changing habits, abnormal activities or health deterioration. Such information has to be communicated to caregivers, doctors, or predefined members of the user’s social circle. In addition to the assisted living and elderly care application, motion detection solutions are frequently used for intrusion detection, crowd estimation, smart-home applications, military and police operations, disaster rescue, and occupational safety systems in industrial areas.

In the last decade, extensive research on device-free localization (DFL) motion and activity detection solutions was carried out using different technologies. The active solutions, based on GPS, radio frequency (RF), ultrasonic, and infrared systems, rely on carrying or attaching a suitable device to the tracked entity, which may cause physical discomfort. DFL solutions detect and track an entity without it carrying any device or participating actively in the process. The entity is detected indirectly by observing changes in the monitored environment. In this respect, different sensor technologies can be utilised, with the number of required devices depending on the application constraints. Optical cameras, passive infrared (IR) sensors, acoustic and vibration sensors, ultrasound, carbon dioxide-based occupancy detection, and RF technology are the most frequently used DFL technologies [1]. Recently, widely studied RF-based DFL systems, first introduced in [2], are exploiting changes in radio signals across different environments in order to detect the entities, since the motion of humans and objects continuously changes magnitude of the scattered components of the wireless signal. Those RF signals can penetrate through the obstacles and walls, which can also result in lower number of sensors required and more loose deployment planning compared to the non-RF-based DFL systems.

In this paper, focus on RF-based motion detection systems, and in particular on their subset referred to as passive device-free systems, where a monitored person does not need to carry any device. However, these systems depend on less-investigated or still-under-development signal processing or localization algorithms that exploit different characteristics of the considered technology and the radio channel properties. Such solutions must be adaptable to the already-furnished interior of the user’s apartment, and any modification of the indoor environment must be kept to a minimum. The operation of existing solutions is often limited to line-of-sight (LoS) conditions, and extension of the coverage typically requires increased number of installed devices, bringing up the total system cost. In this respect, we make use of an easy-to-install robust motion detection system based on three IEEE 802.15.4 ultra-wideband (UWB) radios providing channel impulse response (CIR) information that is exploited by the newly proposed motion detection algorithm, which works also in non-LoS (NLoS) conditions.

The main contributions of the paper are:

- A UWB radio system for motion detection based on a gateway device with signal processing capabilities and three UWB transceivers;
- A publicly accessible dataset for UWB motion detection including the code reproducing the work described in this paper; the dataset is created for motion detection exploration, development of motion detection algorithms and algorithm evaluation;
- An online adaptive motion detection (OAMD) algorithm, which improves the performance of static, offline, or batch motion detection approach by using the online calculated statistics on derivatives of power delay profile (PDP) differences to actively adapt the motion detection threshold in a short-term sliding window to the changing conditions in the operating environment.

The proposed motion-detection solution has been developed and piloted in the H2020 SAAM project (https://saam2020.eu/, accessed: 20 May 2021) as part of the AL system for unobtrusive monitoring and multimodal coaching of the elderly living in their home environment. Elderly participants from Bulgaria, Austria, and Slovenia have been using the SAAM AL pilot system, which connects them to the caregivers and their social contacts using a common interactive communication interface and provides them with personalized coaching based on the data collected in their apartments [3].

The rest of the paper is organized as follows. Section 2 starts with an overview of related work. Section 3 contains a description of the hardware used for UWB-radio-based motion detection, deployment procedure and evaluation data collection process. Section 4 details the main approaches used for detecting the changes in a UWB communication channel. Section 5 describes the offline batch processing approach for motion detection. In Section 6, an online adaptive motion detection algorithm is proposed as the main contribution of the paper. Performance evaluation of the proposed new OAMD algorithm is presented in Section 7 before the concluding remarks are given in Section 8.

## 2. Related Work

An extensive research work on motion detection and activity monitoring utilizing different radio technologies such as radio-frequency identification (RFID), Wi-Fi, ZigBee, Bluetooth low energy (BLE), FM radio, microwave, and UWB radio; adopting different signal descriptors such as received signal strength indicator (RSSI), channel state information (CSI), Doppler effect, and packet reception ratio (PRR); and positioning approaches and methods (radio tomography imaging (RTI), scene analysis—fingerprinting, triangulation, trilateration, hyperbolic lateration, proximity, dead reckoning, etc.) has been performed over time, resulting in different solutions with different characteristics. The main feature determining the performance of radio positioning systems includes the used technology, which, to a large extent, determines the occupied RF bandwidth and positioning method.

Numerous works exist on indoor device-free positioning systems and technologies. For instance, authors in [4] represent one of the latest comprehensive reviews that includes a compilation of the relevant survey papers published until 2019. In [5], a comprehensive survey focused mainly on device-free solutions proposed in the last ten years in various areas of human activity recognition is published. The existing work is divided into action-based, motion-based, and interaction-based areas and is further divided into sub-topics. Many solutions were proposed for human localization, motion detection, activity, and micro-activity recognition based on Wi-Fi RSSI and CSI. Device-free CSI-based methods outperform RSSI-based approaches in terms of the localization accuracy. The advantage of the CSI is in additional information on fading channel included in amplitude and phase, while RSSI is only the average value of the received signal and is severely affected by multipath effects. Several groups of researchers used Wi-Fi RSSI/CSI for localization [6,7,8,9], motion detection [10,11], people counting [12], activity recognition [13,14,15,16], and gesture recognition [17,18,19]. In the following, the latest and most relevant research work on exploring Wi-Fi RF properties is briefly summarized.

One of the first investigations of the AL DFL system using small varying number of wireless sensor networks (WSN) receivers in an indoor environment of 75 m${}^{2}$ was reported in [20]. The authors used three detection approaches, the classifier (CLAS) approach, the shadow-based radio tomographic imaging (SRTI), and the variance-based radio tomographic imaging (VRTI). In [21], the most suitable DFL methods in terms of detection and tracking accuracy were selected, suitable for human monitoring application for elderly and disabled. An RSSI-based DFL system was proposed that can be used to detect and locate elderly people using RSSI measurements and attenuation-based and variance-based methods. Radio channel characteristics influenced by the daily activities of a home occupant were studied in [22]. From the measured channel transfer function (CTF), a complex channel gain, spectrograms, instantaneous mean Doppler shift, and the instantaneous Doppler spread of the channel were derived to demonstrate the advantages of time-frequency spectrogram-based approaches to the activity recognition. In [23], unobtrusive fall detection system based on a Wi-Fi CSI and one-class support vector machine (SVM) classifier was proposed and evaluated. A system based on CSI of Wi-Fi signal able to recognize six daily activities was described in [16]. It uses a feature extraction method and a sparse representation classification (SRC) algorithm. Different scenarios of human behaviour recognition in residential healthcare based on Wi-Fi CSI are described in [24]. The detection of vital signs through the wall is based on changes in signal phase variation. The activity recognition uses high-resolution Doppler shift analysis combined with principal component analysis (PCA) for feature extraction, and SRC for activity classification, while activity monitoring is based on a cross ambiguity function (CAF). In [25], a method was proposed for detecting entities moving at arbitrary speed. The approach captures the variance of variances of amplitudes of each Wi-Fi CSI subcarrier and employs Hidden Markov Model (HMM) to translate the detection in a probability domain which increases the robustness and achieves precision over 98%. CARM system implemented in commercial Wi-Fi device [26] is based on CSI-speed model and CSI-activity model. For the detection of abnormal activities NotiFi was proposed in [27]. It automatically learns several movement categories for abnormal detection by creating multiple hierarchical Dirichlet processes. In [28], a deep-learning-based device-free activity recognition framework was proposed that is independent from the environment. The increased detection accuracy needed for micro-activity recognition methods was obtained in [29] by extracting amplitude and phase information from Wi-Fi CSI. The unique pattern of each activity is revealed by signal processing while machine learning is applied to recognize micro-activities. In [30], a multiTrack system was developed for simultaneously sensing multiple users and their activities. It tracks the reflected signals by analyzing multiple Wi-Fi links at all 5 GHz channels while performing activity recognition by calculating the similarity to the enrolled user profile using the method based on Euclidean distance. Wang in [31] proposed joint activity and indoor localization framework using Wi-Fi CSI fingerprints and deep learning. The latter was used for monitoring human respiration and daily activities [32]. Phase and frequency components of these Wi-Fi radio bursts combined with CAF processing and a deep transfer network (DTN) are exploited. The system capability was experimentally verified by detecting respiration and classifying human motion such as standing, sitting and falling. Damodaran in [33] proved that by combining standard machine learning algorithms such as SVM and long short-term memory (LSTM) with sophisticated signal analysis techniques (wavelet decomposition), various activities could be detected, including fall detection and counting people in a room. In [34], the CSI dataset obtained by software-defined radio (SDR) and algorithm for motion detection were published. The dataset classification was carried out between standing-up and sitting-down activities, and a Random Forest algorithm correctly detected more than 92% of cases. In [35], a CSI-based device-free human activity recognition (CHDAR) system with Wi-Fi sensing radar was developed. To extract activity duration, an adaptive detection threshold was obtained by kernel density estimation (KDE), while for the classification of the frequency domain, a feature random subspace classifier ensemble method was proposed.

Compared to the widely studied Wi-Fi-based approaches, UWB technology is capable of measuring accurate position up to centimetre level due to its high multipath resolution. There are several survey papers on device-free UWB positioning technologies and their comparison to narrowband RF technologies, methods, algorithms, and implementations [36,37,38], so in the following, only the most relevant work for the device-free motion detection applicable for AL systems is briefly reviewed. In [39], a localization method was introduced based on distributed autonomous UWB nodes where a person was detected based on time variations imposed on measured CIR between the transmitter/receiver (Tx/Rx) pair. A maximum likelihood function is used for defining the range between sensors and a person. The solution was tested in a room by three multistate UWB radar systems. Several other papers explore UWB radar principles, and in particular a IR-UWB radar, for indoor wireless positioning [40], object positioning estimation [41], people counting [42,43], tracking locations and recognizing daily activities [44,45], respiration monitoring [46] and fall detection [47].

UWB radar-based systems are typically very expensive and thus not widely applicable for the AL system. Hence, more affordable UWB Tx/Rx solutions exploiting CSI information have attracted considerable research interest. In [48,49], Kilich proposed a device-free person detection and ranging approach based on experimental measurements using five off-the-shelf UWB devices. It relies on exploiting temporal variations in the received signal. The developed signal model was used to obtain the decision statistics to detect the person and to estimate the delay of the reflected signal from the person for which the ranges were obtained. In [50], two received-signal-strength (RSS) variation-estimation methods based on UWB CIR measurements were developed that are more robust to multipath compared to narrowband received signal strength (NRSS) due to the fine multipath resolution of the UWB signals. The methods were tested by measuring CIR in a self-localized network of 10 Decawave TREK100 UWB evaluation kits, and the results confirmed better localization performance and less human effort compared to the narrowband DFL. The accurate positioning in complex environments was achieved by combining advantages of UWB and inertial measurement units (IMU) in [51]. First, the signal transmission law was obtained by distinguishing LoS and NLoS environment followed by eliminating the NLoS influence using maximum likelihood estimation algorithm, and finally the extended Kalman filter information fusion strategy was used. The accurate ranging information is obtained by fusing UWB and angle information of IMU. Human activity recognition (HAR) using UWB signal CIR measurements and ML classification algorithms (naive bayes, multilayer perceptron (MLP), nearest neighbor, random forest) was proposed in [52]. The experiment shows that simple activities such as standing, sitting, and lying can be recognized more accurately than with the Wi-Fi CSI. To further increase the accuracy of the UWB DFL system, the multipath propagation can be exploited. In [53], the amplitude and the phase information (MAMPI) were extracted from the UWB CIR measurements, and a propagation model was proposed that calculates the effect of the person on the received signal. The system was evaluated by simulations and four sensor nodes with Decawave DW1000 that measure UWB CIR. It was confirmed that lower localization error can be obtained by using phase difference and magnitude. The next step in motion detection, particularly for tracking elderly people, was achieved by Kolakowski [54]. He proposed a DFL hybrid localization algorithm that combines time of arrival (TOA) and RSSI measurements of the UWB and BLE radio interfaces and a method for anchor synchronization that enables re-transmission of UWB synchronization packets by the selected anchor nodes. The system is adaptable to specific service requirements and was tested in the laboratory and in elderly people’s homes. In [55], a self-calibrated UWB DFL and activity detection approach is presented. The automatic system setup is performed by the multidimensional scaling (MDS) algorithm. The distance between nodes was estimated by two-way ranging method, while the location of a person was estimated by RTI. It was revealed that the UWB CIR measurements increase the sensitivity of the system with a low number of nodes.

During the survey of related work, we identified two major drawbacks in the existing research. Firstly, there is lack of publicly available datasets, and secondly, the experimental work should be performed in a more realistic environment. Both drawback are addressed in the present work. The experiments were conducted with devices that were actually deployed at the selected pilot sites, i.e., elderly people apartments, and the datasets used for analysing and verifying the proposed motion detection algorithms have been made publicly available.

## 3. AL Motion Detection System

The proposed AL motion detection system consists of hardware (HW) and software (SW) parts. The hardware part consists of UWB sensing devices and a gateway device with signal processing capabilities needed for the motion detection algorithms. In the following subsections, we provide a description of the AL HW components, the motion detection system deployment procedure, and the dataset-generation procedure.

### 3.1. Hardware Components

In our study, we used an AL motion detection system based on custom-designed UWB radio extension card using an STM32F103 mainstream performance line microcontroller (MCU) and DecaWave DWM1000 UWB radio module. The MCU runs custom UWB communication protocol, which consists of two-way ranging (TWR), channel sounding, and result-reporting capabilities. In the SAAM project, UWB extension cards are used in two types of devices, a standalone UWB device and an edge gateway (EGW) device.

The EGW device is a custom embedded computer based on BeagleCore^™^ single-board computer (SBC) running GNU/Linux. It acts as an internet gateway for all wireless devices deployed in the living environment without their own internet connectivity. The EGW device is powerful enough to perform data collection, signal processing, and feature extraction as well as all data-forwarding tasks. One of the key tasks of EGW in the AL deployment is UWB data collection and signal processing with the activity-detection algorithm implementation.

**Figure 1:**
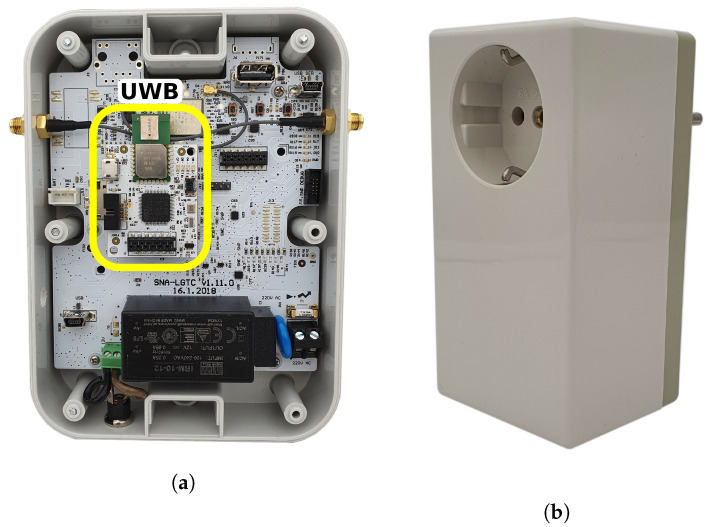
(**a**) UWB extension card integrated in EGW device. (**b**) Standalone UWB socket-onsocket device.

In the proposed solution, UWB radio mounted in the EGW device acts as a master device. It coordinates the communication activities by sending beacons containing requests for slave devices. UWB radios in standalone devices acting as slave devices predominantly listen for beacons received from the master device and respond accordingly [55]. A typical pilot deployment in SAAM consists of two standalone UWB devices and one EGW device. Standalone UWB devices are provided in a socket-on-socket enclosure for easy installation and minimal interference to the existing living environment. This approach ensures unrestricted use of existing sockets while covering the environment at the adequate height from the floor. Examples of an EGW device with mounted UWB card and a standalone socket-on-socket UWB device are presented in Figure 1.

### 3.2. Deployment Procedure and Dataset Generation

**Figure 2:**
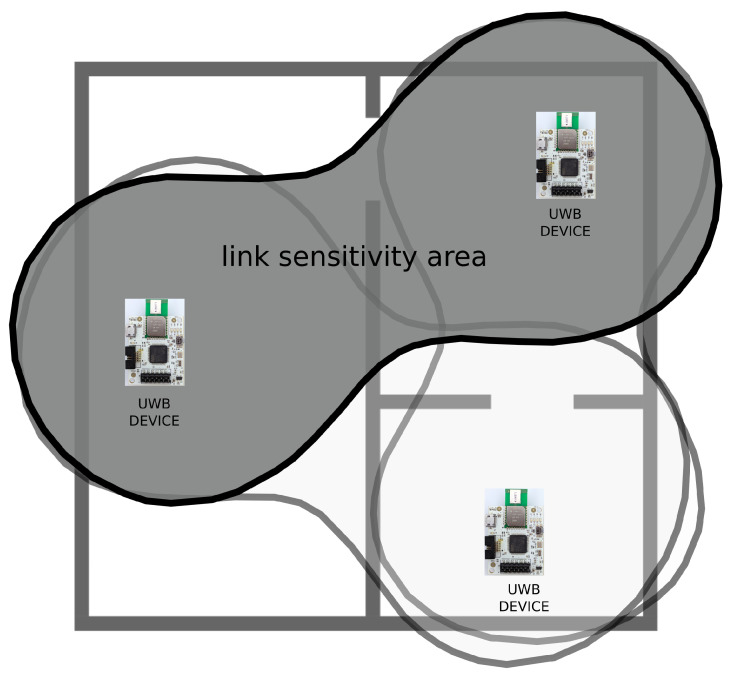
Graphical representation of deployment of three UWB devices in an apartment with link sensitivity areas visualized by Cassini oval shapes.

In order to maximize the motion detection area in the apartment, the radio links have to be organized in a way to maximize the area covered by the links. The main condition for a moving target to be detectable by a TX-RX pair is that it must be inside a maximum Cassini oval whose size depends on the antenna gains, transmitted power, wavelengths, and receiver’s signal-to-noise ratio (SNR) [56]. For instance, in the SAAM project with three UWB devices per pilot site, the maximum coverage is obtained when the deployed UWB devices resemble triangular shape, as shown in Figure 2. The size of the apartment that can be covered by three UWB transceivers, or the distance in indoor environment that can be covered by one pair of UWB devices, strongly depends on the actual environment layout, the number and type of walls between the devices, and the furniture layout. Exact locations of devices may be further conditioned by the positions or availability of power sockets, so in most cases, the resulting links between devices are characterized as NLoS. Nonetheless, in our testing deployments, we successfully established a link across more than 12 m with four intermediate internal plaster walls, whereas in outdoor LoS conditions, according to the device specification, a communication can be established between a pair of UWB devices operating at maximum permitted output power at a distance of up to 290 m at 10% packet error ratio (PER) [57].

To conduct quality research and fair performance comparison with existing solutions, the availability of a representative and suitably documented dataset is of particular importance. Thus, in order to collect data, we equipped three different indoor environments with individual motion detection systems each consisting of three UWB devices. The first setup consisted of three UWB nodes placed in a $\;25\;m^{2}$ office environment with LoS links between the UWB pairs. The second setup was deployed in a small living apartment of $\;20\;m^{2}$ with similar layout to the one depicted in Figure 2, where internal walls of plaster and bricks as well as furniture prevented establishing LoS UWB links between all UWB pairs. The third setup was deployed for an additional validation step in a different $\;40\;m^{2}$ indoor environment with all three UWB radio links in NLoS configuration, where LoS of one link was blocked by a concrete wall and the other two by the furniture.

Each motion detection system continuously recorded CIR for all three radio links in the corresponding indoor environment. The effective CIR sampling frequency on each radio link was approximately $f_{s}=7.5\;\mathrm{Hz}$, determined by the processing capabilities of UWB devices and the protocol for collecting CIR measurements. Actual sampling frequency recorded in a dataset can slightly deviate due to random errors encountered on the universal asynchronous receiver-transmitter (UART) interface used for data capturing at 921,600 baud rate.

The representative motion dataset was generated during all three separate measurement campaigns. The first measurement campaign covered channel sounding recordings in both indoor environments without strict activity labelling. Thus, data from this campaign do not include any time stamps or labels for motion events, only time stamps when the environment became occupied and when it was emptied. This part of the dataset was used for subsequent statistical analysis and algorithm development and contains seven longer-term recordings in office environment with recording time of 482 h or 20.1 days. The second measurement campaign covered channel sounding recordings that were collected for creating a dataset for evaluation of motion detection. During the activity-labelling measurement campaign, a person at the monitored location manually logged all activities with the corresponding time stamps. The validation part of the dataset contains three validation recordings from the office environment and 33 validation recordings from the small apartment environment with the cumulative recording time of 45 h. The third measurement campaign provided an additional validation dataset containing 42 validation recordings with cumulative time of 27 h. The combined motion dataset contains approximately 23 days of raw recordings from all measurement campaigns.

The collected dataset used in this paper and the corresponding code used for data preparation, data processing, and performance analysis are freely available to enable replication and continuation of work described [58]. For more interactive experience and easy tweaking of the parameters used in the algorithms, the code was written using Python programming language in a form of Jupyter notebooks.

## 4. Detecting Changes in Radio Channel

RF-based, device-free localization and motion detection systems exploit the variability of the radio channel properties caused by moving obstacles. The moving obstacle may shadow the first Fresnel zone in direct line between a transmitter and a receiver (LoS link) and thus introduce additional attenuation of the radio signal. This additional attenuation of the received signal is exploited in a concept of the RF tomography approach observing the RSS between several radio links.

In living indoor environments, it is difficult to achieve a sufficient number of LoS links with reasonable number of RF sensing devices for localization and motion detection. However, in the indoor environments, we can take advantage of multipath radio propagation considering radio signal reflections as virtual radio links extending the coverage outside the first Fresnel zone, thus lowering the actual number of physical radio links needed for a sufficient coverage of the sensitivity area. The size of the sensitivity area depends on the characteristics of the propagating environment [59]. In indoor environments, it is generally larger than outdoors because of more pronounced multipath effects. While the radio signal reflections cannot be distinguished in narrow-band radio technologies, in the wide/broad-band wireless communications, the multipath reflection can be estimated by measured PDP and CIR. Any obstacle in the direct LoS path and reflected path will impact the CIR.

**Figure 3:**
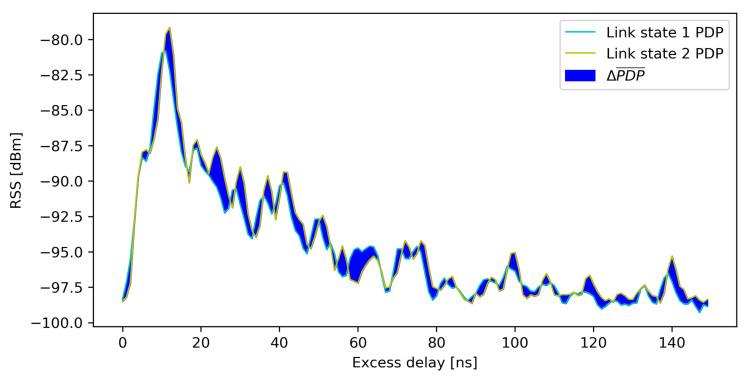
Two PDPs for different states of the same radio link. Link state 1 PDP presents the average PDP for one radio environment state. Link state 2 PDP presents the average PDP for another state of radio environment, where a person changed the location within the same environment. The difference between two states is presented with absolute PDP difference marked in blue.

Figure 3 shows the approach to extract and detect changes in the monitored environment using the provided CIR information from the deployed UWB system. The changes in the radio environment are reflected in CIR. For example, if a person moves to a new position and the motion shadows a new radio path, a CIR is changed. The path length of the reflected ray is always longer than path length of the direct ray, so the multipath CIR components following the first path CIR component are more affected when the changes happen outside the first Fresnel zone. For more convenient representation and further calculation, as well as better clarity, the CIR information is represented as PDP in dBm.

### 4.1. Power Delay Profile Moving Average

In the DW1000 UWB radio, the CIR accumulator has 992 bins for the nominal 16 MHz mean pulse repetition frequency (PRF) and 1016 samples for the nominal 64 MHz mean PRF, respectively. Part of the CIR accumulator before the first path index, which designates the detected start of received UWB pulse, contains only noise. The useful length of CIR (i.e., the part of CIR containing actual CIR data) is typically around 152 bins, which was selected as a base CIR length T=152 for all presented research activities.

CIR measurements are very noisy and are not suitable for direct calculation of PDP differences in consecutive CIR samples (sampling period in our case equal to approximately 133 ms). Based on the assumption that changes in CIR are caused by changes in the environment due to movement of people, furniture, or other obstacles and are slow compared to noise frequencies, the moving average of collected PDPs can be used to reduce the PDP noise level. To make the values more clear and convenient for presentation and further calculation, raw unitless CIR accumulator values obtained from the DW1000 UWB radio are transformed to PDP using Equation (1) [60]:

(1) $$\begin{array}{cc} \mathrm{PDP} & =\left[p_{0},p_{1},\ldots p_{i},\ldots p_{T}\right],\;T=152\\ p_{i} & =10\times \log_{10}\left(\frac{V_{i}\times 2^{17}}{N^{2}}\right)-121.74\;\left[d\mathrm{Bm}\right], \end{array}$$

where $p_{i}$ is power of the PDP at the *i*-th bin, $V_{i}$ is the value of the input CIR signal in the *i*-th bin, *N* is the number of received preamble symbols in the CIR accumulator, and *T* is the number of bins in resulting PDP extracted from the entire CIR accumulator. For easier and more convenient calculations, PDPs are transformed from logarithmic dBm units to linear mW units by

(2) $$\mathrm{PDP}\;\left[mW\right]=10^{\frac{\mathrm{PDP}[d\mathrm{Bm}]}{10}}.$$

Then, the average PDP signal, $\bar{\mathrm{PDP}}$, is calculated by element-wise averaging of a sequence of *M* PDPs as

(3) $$\bar{\mathrm{PDP}}=\frac{1}{M}\sum_{i=1}^{M}{\mathrm{PDP}}_{i}.$$

### 4.2. PDP Differences

The difference between two average PDPs, ${\bar{\mathrm{PDP}}}_{j}$, and ${\bar{\mathrm{PDP}}}_{j-1}$, corresponds to the amount of change in the radio environment that occurs between those two time instances. The first average PDP, ${\bar{\mathrm{PDP}}}_{j}$ contains M most recent PDP measurements (i∈[1,M]) and the second average PDP, ${\bar{\mathrm{PDP}}}_{j-1}$, contains the previous M PDP measurements (i∈[M+1,2M]). The difference $\Delta {\bar{PDP}}_{j}$ is calculated by element-wise subtraction of two PDP moving averages ${\bar{\mathrm{PDP}}}_{j}$ and ${\bar{\mathrm{PDP}}}_{j-1}$ and summation of elements as

(4) $$\Delta {\bar{PDP}}_{j}=∥{\bar{\mathrm{PDP}}}_{j}-{\bar{\mathrm{PDP}}}_{j-1}∥.$$

In Equation (4), ${\bar{\mathrm{PDP}}}_{j}$ and ${\bar{\mathrm{PDP}}}_{j-1}$ are PDP moving averages that hold the moving averages of subsequent PDP measurements (e.g., in our case, ${\bar{\mathrm{PDP}}}_{j}$ holds the moving average of the most recent 100 PDPs in the stream and ${\bar{\mathrm{PDP}}}_{j-1}$ holds the moving average of the previous 100 PDPs in the stream).

**Figure 4:**
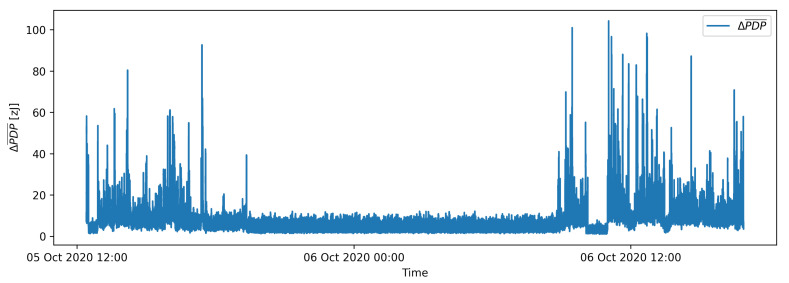
Time series of PDP differences for a 30-h long recording in a pilot environment.

The resulting values are the numerical integrals of absolute differences between two moving averages and describe the difference in average energy received per one UWB symbol in zeptojoules (zJ). Figure 4 shows time series of calculated PDP differences ($\Delta \bar{PDP}$) on measurements of one link in an office environment in a thirty-hour period. The flat part of the presented signal between 5 October 2020 at 21:00 and 6 October 2020 at 9:00 present $\Delta \bar{PDP}$ signal for an empty environment, preceded and succeeded by a signal denoting significant movement events.

**Figure 5:**
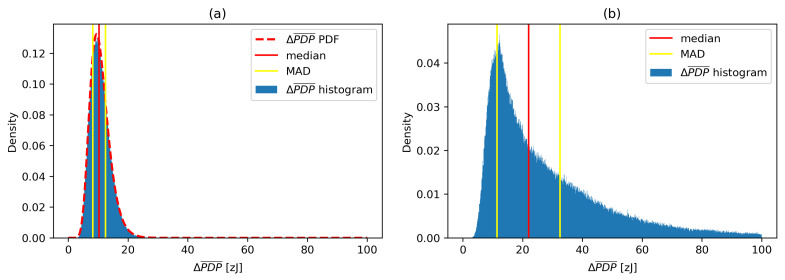
$\Delta \bar{PDP}$ histograms for the environment without (**a**) and with (**b**) movement.

A $\Delta \bar{PDP}$ histograms for multi-day channel sounding measurements in the same environment are presented in Figure 5 without any significant movement (a) and with intensive movement (b), respectively. The probability distributions of $\Delta \bar{PDP}$ signals have median values of $10.3\times 10^{-21}\;J$ and $22.0\times 10^{-21}\;J$, respectively, with median absolute deviations around median (MAD) at $2.13\times 10^{-21}\;J$ and $10.52\times 10^{-21}\;J$, respectively, indicating large variations of channel conditions caused by the movement in the operating environment. We have selected MAD as a robust measure of statistical dispersion due to its larger resilience to outliers in a dataset than the standard deviation.

## 5. Batch Processing Motion Detection

A stream of $\Delta \bar{PDP}$ data in Figure 4 shows that the $\Delta \bar{PDP}$ values increase when motion emerged in the environment and return to a steady state when the motion is no longer present. One example of a steady state is presented in Figure 4 between 5 October 2020 at 21:00 and 6 October 2020 at 9:00, where signal fluctuation represents noise of an empty environment mostly due to the changes in propagation conditions in the selected stationary environment during the operation as well as changes in the performance of the radio equipment. It can be intuitively assumed that an appropriate threshold can be selected to determine whenever there is or is not any motion in the monitored environment. However, according to the evaluation of actual long-term data included in the dataset and all labelled examples, the motion detection based on a fixed threshold $\Delta \bar{PDP}$ showed very limited performance.

In particular, we first used the batch processing approach to motion detection where we calculated median and MAD values for a batch of past N PDP differences $\Delta {\bar{PDP}}_{j}$ and determined a suitable fixed motion detection threshold $Thr_{l}$ expressed as the number of *k* MAD values away from the median

(5) $$Thr_{l}=median\left(\Delta {\bar{PDP}}_{j}\right)+k\times MAD\left(\Delta {\bar{PDP}}_{j}\right),\;where\;j\in \left[l-N,l\right].$$

**Figure 6:**
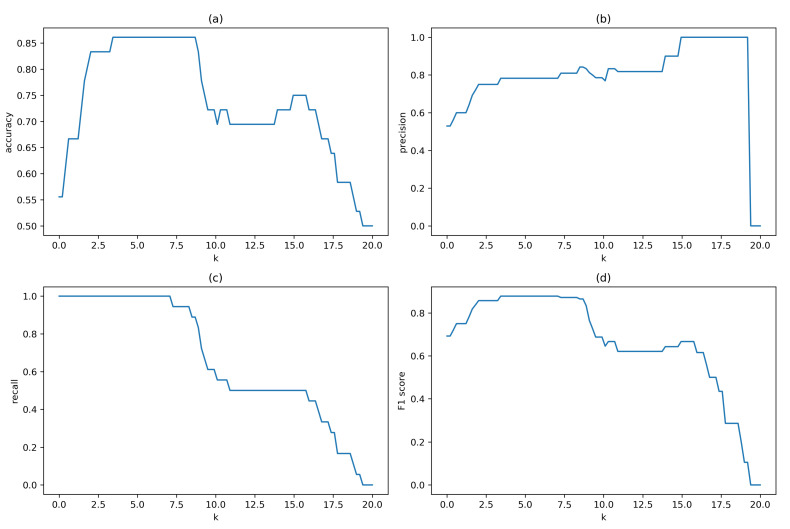
(**a**) accuracy, (**b**) precision, (**c**) recall and (**d**) F1 score graphs presenting classification performance of batch-processing motion detection in dependency of increasing threshold.

We investigated the range of most suitable values for *k* on one of the validation datasets from the office environment with manually annotated motion events. We evaluated the classification performance in terms of accuracy, precision, recall, and F1 score. Results for these performance metrics versus increasing threshold as the number *k* of MAD values from 0 to 20 in step by 0.2 are depicted in Figure 6, indicating that best performance for this batch was obtained for *k* between 3 and 7. Results for k=5 are summarised in Table 1 and show that the samples were accurately classified in 86% of cases, with 78% precision for activity samples and 100% for static samples. Recall statistics present a positive bias towards detecting activity samples with 100% recall and less reliable detection of static samples with 72% recall.

**Figure 7:**
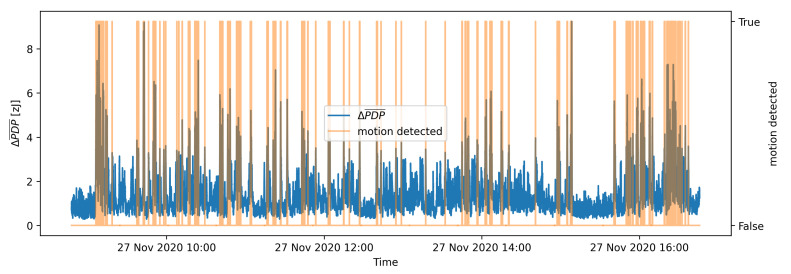
Results of the batch processing motion detection algorithm with k=5 for a part of validation data set.

Time series of $\Delta \bar{PDP}$ for the validation dataset and the results of the batch-processing motion detection approach for k=5 are shown in Figure 7. This approach has several drawbacks. Firstly, it requires large amount of storage space and memory for storing long-term batch of measurements that is usually not available on resource-limited edge devices. Secondly, the motion can be detected only for past events and therefore cannot be used as online motion detection system. Finally, the motion detection performance is based on the statistics of the batch, and the most suitable value of *k* for motion detection threshold needs to be determined for each batch separately. In the following performance comparison, we used k=5.

## 6. Online Adaptive Motion Detection Approach

To overcome the problems regarding the storage and memory usage and to enable online (i.e., instant) motion detection, we propose a new algorithm with online adaptive motion detection threshold within a sliding window to identify motion samples in $\Delta \bar{PDP}$ values.

Results in the previous section demonstrated that raw $\Delta \bar{PDP}$ values are not suitable for calculation of the motion detection threshold, because the values influenced by the presence of motion that are mostly in the tail of the $\Delta \bar{PDP}$ distribution increase the threshold values, which in turn decreases the algorithm’s sensitivity to motion. To eliminate this effect, we use the statistics of the first derivatives of $\Delta \bar{PDP}$ values, denoted as $\Delta^{2}\bar{PDP}$ and representing change in motion, for adapting threshold values. These derivatives describe the fluctuations between successive $\Delta \bar{PDP}$ values and are more suitable for online threshold values.

In the following, we first look at the statistics and define models of the derivative of $\Delta \bar{PDP}$ values, i.e., $\Delta^{2}\bar{PDP}$, for online threshold estimation. Then, we define the scaling procedure for estimating the motion detection thresholds based on $\Delta^{2}\bar{PDP}$ statistics, and lastly, we define the entire motion detection algorithm.

### 6.1. Adaptive Threshold Estimation

**Figure 8:**
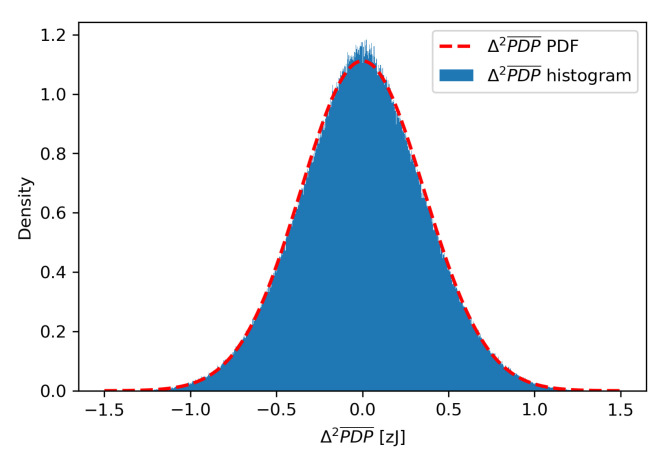
$\Delta^{2}\bar{PDP}$ histogram and probability distribution.

An estimated value for $\Delta^{2}\bar{PDP}$ can be obtained by subtracting consecutive $\Delta \bar{PDP}$ values, which is described by Equation (6). Figure 8 presents a histogram of $\Delta^{2}\bar{PDP}$ values for multi-day channel sounding measurements in an environment without any significant movement. The histogram resembles normal distribution, and normality of $\Delta^{2}\bar{PDP}$ probability distribution was confirmed with the D’Agostino’s $K^{2}$ test yielding *p* = 0.891. The mean value of the $\Delta^{2}\bar{PDP}$ and its standard deviation are equal to $\mu_{N}=-1.0\times 10^{-27}\;J$ and $\sigma_{N}=3.59\times 10^{-22}\;J$, respectively.

(6) $$\Delta^{2}{\bar{PDP}}_{j}=\Delta {\bar{PDP}}_{j}-\Delta {\bar{PDP}}_{j-1}$$

We expect $\Delta \bar{PDP}$ data samples caused by motion to represent outliers in the range of or exceeding maximum values of their distribution obtained in an environment with no motion. This means that an online adaptive motion detection threshold should follow the envelope of maximum values in a sliding window for an environment without motion.

**Figure 9:**
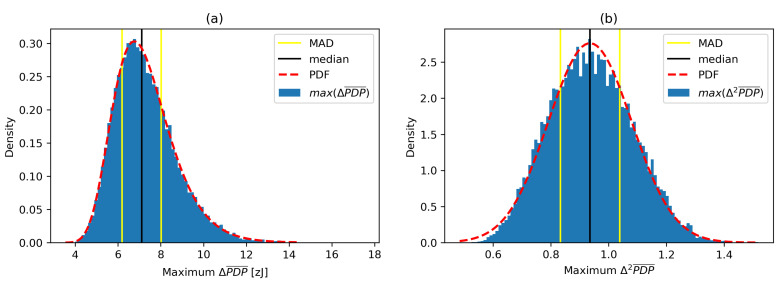
Histograms and probability distributions of windowed (**a**) maximum $\Delta \bar{PDP}$ values and (**b**) maximum $\Delta^{2}\bar{PDP}$ values.

To estimate the statistics of maximum windowed $\Delta \bar{PDP}$ values and maximum windowed $\Delta^{2}\bar{PDP}$ values, we applied a sliding window with the window length $w_{l}=100$ on the selected *N* samples from the environment without motion. This gives $N-(w_{l}-1)$ maximum $\Delta \bar{PDP}$ and $\Delta^{2}\bar{PDP}$ estimates. Histograms, fitted probability density functions and some statistical values for online maximum $\Delta \bar{PDP}$ and online maximum $\Delta^{2}\bar{PDP}$ values in a sliding window are depicted in Figure 9.

Median and MAD of online maximum $\Delta \bar{PDP}$ values are $median_{\Delta max}=7.11\times 10^{-21}\;J$ and $MAD_{\Delta max}=0.92\times 10^{-21}\;J$, respectively. Median and MAD of online maximum $\Delta^{2}\bar{PDP}$ values are $median_{\Delta^{2}max}=0.94\times 10^{-21}\;J$ and $MAD_{\Delta^{2}max}=0.10\times 10^{-21}\;J$, respectively.

A particular online maximum $\Delta^{2}\bar{PDP}$ value in a selected window is obtained from the last $w_{l}$$\Delta^{2}\bar{PDP}$ samples, so it estimates the maximum fluctuation level of the most recent $w_{l}$$\Delta \bar{PDP}$ samples in motionless environment. The estimated online maximum $\Delta^{2}\bar{PDP}$ value has suitable time dependence for a motion detection threshold, but it needs to be scaled up to the range of $\Delta \bar{PDP}$ values. We propose a scaling factor $k_{max}$, which is defined as a ratio of median value of maximum windowed $\Delta \bar{PDP}$ probability distribution, $median_{\Delta max}$, and online median value of maximum windowed $\Delta^{2}\bar{PDP}$ probability distribution, $median_{\Delta^{2}max}$.

(7) $$k_{max}=\frac{median_{\Delta max}}{median_{\Delta^{2}max}}$$

Similarly, a particular online $\Delta^{2}\bar{PDP}$ MAD value is estimated based on the selected window including the last $w_{l}$$\Delta^{2}\bar{PDP}$ samples. This estimates the current deviation of the process based on the most recent $w_{l}$$\Delta \bar{PDP}$ samples. The estimated MAD of $\Delta^{2}\bar{PDP}$ can be scaled up to the range of $\Delta \bar{PDP}$ MAD values using a scaling factor $k_{MAD}$, which is defined as a ratio of MAD of windowed maximum $\Delta \bar{PDP}$ values, $MAD_{\Delta max}$, and MAD value of online windowed maximum $\Delta^{2}\bar{PDP}$ values $MAD_{\Delta^{2}max}$. This parameter scales the online median absolute deviation of $\Delta^{2}\bar{PDP}$ in the range suitable for motion detection threshold.

(8) $$\begin{array}{cc} k_{MAD} & =\frac{MAD_{\Delta max}}{MAD_{\Delta^{2}max}} \end{array}$$

Multiplication factors $k_{max}$ and $k_{MAD}$ were defined based on the windowed maximum $\Delta \bar{PDP}$ and maximum $\Delta^{2}\bar{PDP}$ histogram-based models on a dataset of four recording sessions witha total of 17.7 days (424 h) of cumulative recording time. The final values of factors $k_{max}$ and $k_{MAD}$ were defined by calculating the mean of four values $k_{maxi}$ and four values of $k_{MADi}$, respectively. The resulting scaling factors are equal to $k_{max}=7.451$ and $k_{MAD}=10.980$.

### 6.2. Online Adaptive Motion Detection Algorithm

**Figure 10:**
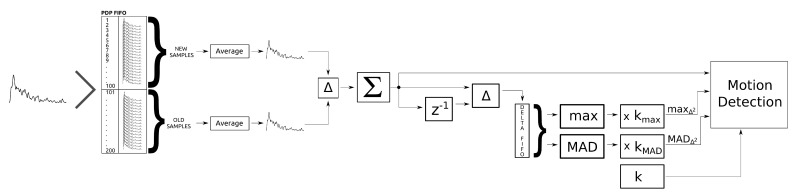
Online adaptive motion detection algorithm flowchart.

The complete OAMD algorithm is schematically presented in Figure 10. The radio channel is periodically sampled for CIR and subsequently transformed to PDP expressed in mW. Each new PDP is put into the PDP first-in-first-out (FIFO) buffer with a fixed length that holds the most recent 200 PDP samples. The PDP FIFO buffer is split into two parts with 100 PDP samples each; 100 older PDP samples and 100 newer PDP samples. By each newly included PDP sample, two moving average PDPs are calculated based on 100 older PDP samples, ${\bar{\mathrm{PDP}}}_{j-1}$, and 100 newer PDP samples, ${\bar{\mathrm{PDP}}}_{j}$, respectively, each corresponding to approximately 13.3 s of short-term history. Next, a differential PDP sample is calculated between ${\bar{\mathrm{PDP}}}_{j}$ and ${\bar{\mathrm{PDP}}}_{j-1}$ at each PDP update, and its absolute difference summed into a $\Delta \bar{PDP}$ value.

The difference between the new and the previous $\Delta \bar{PDP}$ value (i.e., $\Delta^{2}\bar{PDP}$) is calculated and inserted in the fixed length delta FIFO buffer holding the most recent 100 $\Delta^{2}\bar{PDP}$ values. For each PDP update, the maximum $\Delta^{2}\bar{PDP}$ value, $max_{\Delta^{2}}$, and MAD, $MAD_{\Delta^{2}}$, are calculated for the values in delta FIFO buffer and multiplied by the corresponding scaling factors $k_{max}$ and $k_{MAD}$. Finally, the current $\Delta \bar{PDP}$ value is used, together with the scaled $max_{\Delta^{2}}$ and $MAD_{\Delta^{2}}$ values, as an input to the motion detection algorithm.

**Figure 11:**
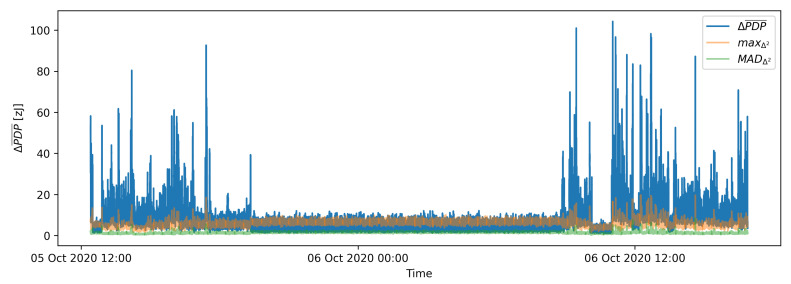
Multiplied maximum $\Delta^{2}\bar{PDP}$ and multiplied standard deviation of maximum $\Delta^{2}\bar{PDP}$ against $\Delta \bar{PDP}$.

Figure 11 shows the $\Delta \bar{PDP}$ signal as well as scaled $max_{\Delta^{2}}$ and $MAD_{\Delta^{2}}$ signals. It is evident that the $max_{\Delta^{2}}$ and $MAD_{\Delta^{2}}$ values follow the $\Delta \bar{PDP}$ envelope.

**Figure 12:**
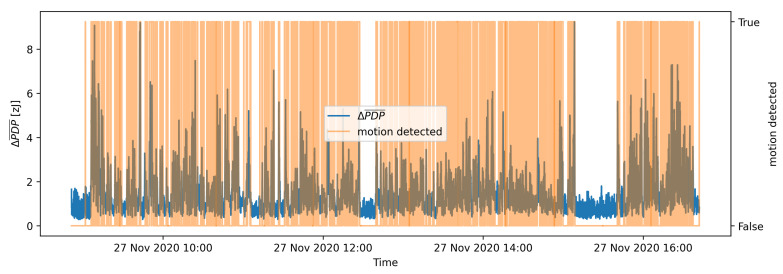
Results of the OAMD algorithm with *k* = 5 for a part of the validation data set.

Figure 12 presents the results of OAMD motion detection on the same data that were used in Figure 7 for the batch process motion detection. Figure 7 and Figure 12 indicate that the adaptive detection threshold in OAMD algorithm helps detecting the motion considerably more reliably also in highly dynamic conditions, whereas batch processing motion detection becomes rather insensitive in an environment characterised by significant motion. Flat sections of the $\Delta \bar{PDP}$ graph are detected as motionless environment, and parts of $\Delta \bar{PDP}$ graph with higher $\Delta \bar{PDP}$ values are recognized as motion presence. The motion detection graph marks the $\Delta \bar{PDP}$ values with orange at the moments where motion was detected. The actual value of the motion detected is boolean True or 1 and boolean False or 0 for the moments, where motion was not detected. A quantitative evaluation of the OAMD performance is discussed in the following section.

## 7. Performance Evaluation

To efficiently evaluate the performance of the OAMD algorithm, we prepared a validation dataset based on the recorded data as described in Section 3.2. Thus, nine records with activity tags and 45 h of cumulative recording time were used for the system evaluation. The excerpts of data were prepared based on the annotations made during the recording sessions. Each data excerpt contains the data between two time stamps describing particular activity in the environment and additional 1000 samples before the starting time stamp and 1000 samples after the end of the activity time stamp. The extra samples are included to fully initialize the motion detection algorithm and fill FIFO buffers in order to get credible detection results throughout the entire activity interval.

Activity samples were initially divided into three groups: activity, sporadic activity, and static environment. The activity group contained a few short events describing certain motion activity. The sporadic activity group contained the data excerpts describing longer periods of time containing sporadic activity including motion. The activity bursts were present, but they lacked the specific time stamps of particular motion activities. The last group of samples contained the excerpts with no motion activities; i.e., they correspond to static environment. For classification purposes, we combined the first two groups into one larger activity group containing 18 data excerpts. In order to balance the activity group, we also created a larger static group containing 18 excerpts from the group with no activity.

The evaluation process was defined as a classification problem where each data excerpt was analyzed by the OAMD algorithm. If the algorithm detected motion at any part of the data excerpt, the data excerpt was classified as an activity sample. When motion detection algorithm did not detect any activity, the data excerpt was classified as a static sample. Comparing the actual labels of the data samples and classification results enabled compiling classification statistics for performance evaluation of the OAMD algorithm.

As was shown in Section 5, the motion detection performance is heavily influenced by the current statistics of raw $\Delta \bar{PDP}$ measurement values. Therefore, as shown in Table 1, the resulting motion detection statistics for the batch-processing motion detection algorithm did not show a very reliable performance. On the contrary, the proposed OAMD algorithm showed an excellent adaptability to all environmental conditions contained in the evaluation dataset. The results in Table 2 show perfect motion detection performance. It can be expected that OAMD motion detection performance will slightly deteriorate in different environments. However, since no dataset can cover all possible situations that the system can encounter during the real-life operation, the deterioration is not expected to be significant.

To test this hypothesis, we used the additional set of validation measurements from a different, bigger, 40 m${}^{2}$ indoor living space environment, as described in Section 3.2. The samples recorded in a stretch of 27 h included 21 validation excerpts with no activity and 21 validation excerpts with activity present in the environment. The resulting activity detection performance in the second validation environment deteriorated a bit with the overall accuracy of 98%. A closer examination showed that the OAMD algorithm falsely detected activity in only one sample within an excerpt of empty environment with the length of 2 h and 45 min. The complete classification statistics results are presented in Table 3.

**Figure 13:**
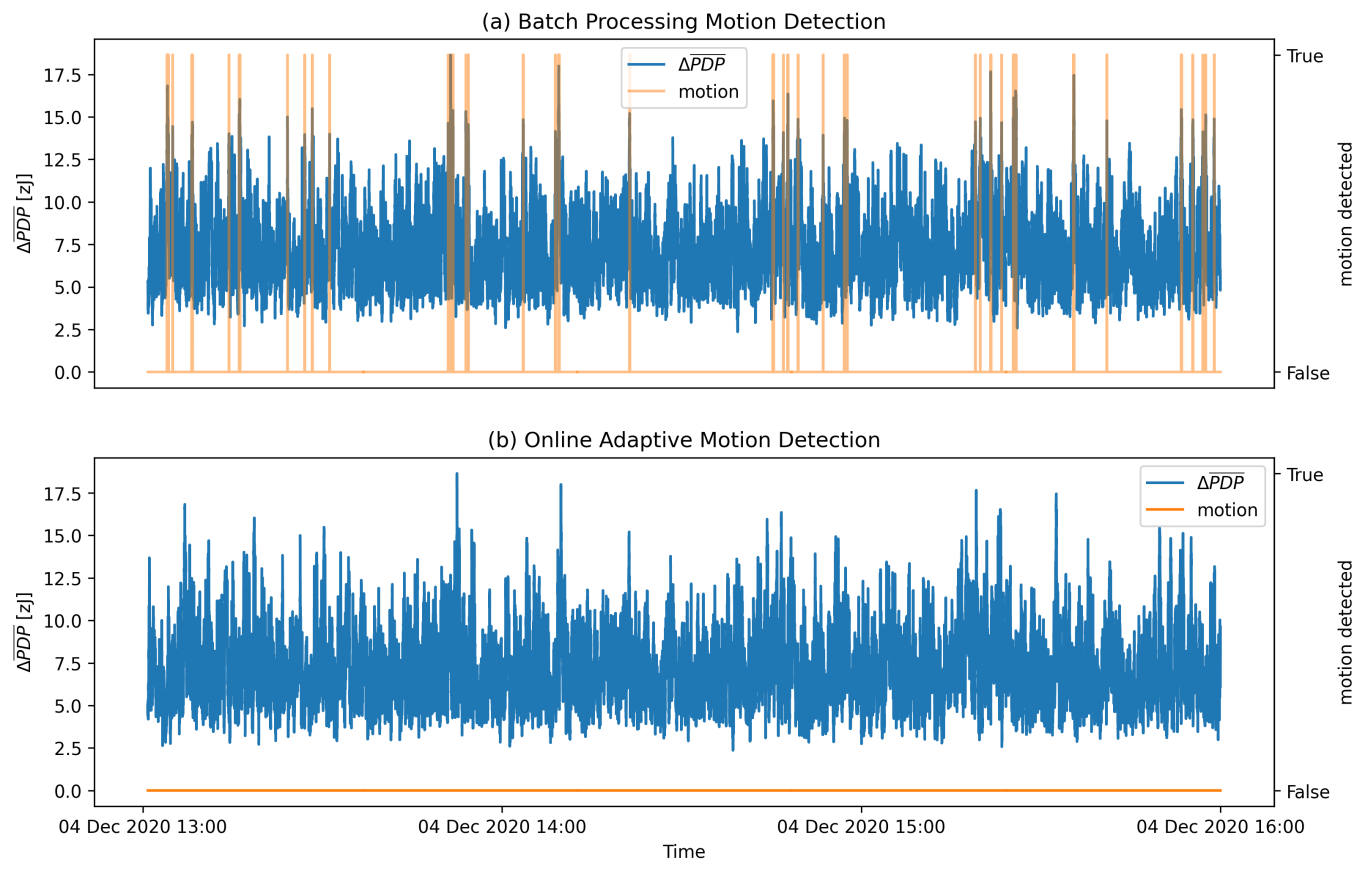
Comparison of motion detection performance of (**a**) batch-processing motion detection and (**b**) OAMD algorithms on identical data stream representing static environment.

A comparison of motion detection performance of both evaluated algorithms on the same validation data is presented in Figure 13. The data sample used represents a static environment with no activity. Hence, it is expected that the motion detection algorithm should not detect any motion. The proposed OAMD algorithm shows perfect motion detection performance, whereas batch-processing motion detection algorithm exhibits many false positive decisions.

## 8. Conclusions

In this paper, we addressed the issue of wireless device-free motion detection in typical indoor spaces by exploiting the changes in the channel impulse response of the UWB radio channel, which indicates the motion activity of people or/and objects within the monitored environment. The main motivation for developing the solution is extending independent living of the elderly in their homes.

The solution is based on IEEE 802.15.4 UWB CIR data which, are periodically measured for all communication links within the monitored environment. The measurements are temporarily stored to produce two delayed moving average PDP envelopes further used for calculating a scalar PDP difference, $\Delta \bar{PDP}$. To avoid the influence of raw PDP differences on the motion detection threshold, we proposed an approach that adapts the threshold to the scaled-up statistics of the first derivatives of PDP differences, $\Delta^{2}\bar{PDP}$, representing changes in motion. The proposed online adaptive motion detection algorithm uses online maximum value and median absolute deviation of 100 most recent $\Delta^{2}\bar{PDP}$ values and scales them to adequate level using scaling factors $k_{max}$ and $k_{MAD}$.

The main advantage of the proposed OAMD algorithm is in the ability to adapt the detection threshold according to the fluctuations of the signal in an environment with no motion. The resulting adaptive threshold depends only on the variability of the measurement equipment and long-term changes in the propagation environment, thus making the OAMD algorithm very reliable. The algorithm is able to detect small changes in the environment both in LoS and NLoS conditions. Although the sensitivity is lower in NLoS conditions, the coverage compared to the optical approaches relying on LoS conditions is significantly extended. In comparison to the radar-based solutions, which can provide additional metrics such as velocity, direction of movement, etc., the proposed approach is cheaper to produce and deploy, which is essential for the target users in AL domain.

As part of future research work, we are planing to extend the datasets by recording more activities at different locations. With more detailed samples, the sensitivity of the system to the level and type of motion in the environment can be analyzed. In particular, motion sensitivity will be analyzed by controlling the level of motion and the position of moving person or object relative to the measurement equipment. Moreover, current trends in time series classification and anomaly or event detection research provide an interesting ground for investigation of alternative approaches to threshold estimation and motion detection by using self-supervised learning approaches, convolutional neural networks (CNN), LSTM, or other recurrent neural network (RNN) architectures and reinforcement learning techniques.

## Figures and Tables

**Table 1:** Classification performance for batch processing motion detection for the selected dataset and fixed threshold at 5 × MAD.

Class	Precision	Recall	F1 Score	Support
Activity	0.78	1.0	0.88	18
Static	1.0	0.72	0.84	18
			**Accuracy**:	**0.86**

**Table 2:** Classification statistics for OAMD algorithm.

Class	Precision	Recall	F1 Score	Support
Activity	1.0	1.0	1.0	18
Static	1.0	1.0	1.0	18
			**Accuracy:**	1.0

**Table 3:** Classificationstatistics in the second validation environment for OAMD algorithm.

Class	Precision	Recall	F1 Score	Support
Activity	0.95	1.0	0.98	21
Static	1.0	0.95	0.98	21
			**Accuracy:**	0.98

## Data Availability

Not applicable.

## Abbreviations

The following abbreviations are used in this manuscript:

AL	assisted living
BLE	Bluetooth Low Energy
CAF	cross ambiguity function
CHDAR	CSI-based device-free HAR
CIR	channel impulse response
CLAS	classifier
CNN	convolutional neural network
CSI	channel state information
CTF	channel transfer function
DFL	device-free localization
DTN	Deep Transfer Network
EGW	edge gateway
FIFO	first-in-first-out
FM	frequency modulation
GPS	Global Positioning System
HAR	Human Activity Recognition
HMM	hidden Markov model
IEEE	Institute of Electrical and Electronics Engineers
IMU	inertial measurement unit
IR	infrared
KDE	kernel density estimation
LoS	line-of-sight
LSTM	long short-term memory
MCU	microcontroller unit
MDS	multidimensional scaling
ML	machine learning
MLP	multilayer perceptron
NLoS	non-line-of-sight
NRSS	narrowband received signal strength
OAMD	online adaptive motion detection
PCA	principal component analysis
PDF	probability density function
PDP	power delay profile
PHY	physical layer
PIR	passive infrared
PRF	pulse repetition frequency
RF	radio frequency
RFID	radio-frequency identification
RNN	recurrent neural network
RSS	received signal strength
RSSI	received signal strength indicator
RTI	radio tomographic imaging
SBC	single-board computer
SDR	software-defined radio
SNR	signal-to-noise ratio
SRC	sparse representation classifier
SRTI	shadow-based radio tomographic imaging
SVM	support vector machine
TOA	time of arrival
TWR	two-way ranging
UART	universal asynchronous receiver-transmitter
UWB	ultra-wideband
VRTI	variance-based tomographic imaging
WSN	wireless sensor networks
